# Assessment of vitamin D and its association with cardiovascular disease risk factors in an adult migrant population: an audit of patient records at a Community Health Centre in Kensington, Melbourne, Australia

**DOI:** 10.1186/1471-2261-14-157

**Published:** 2014-11-11

**Authors:** Thilanga Ruwanpathirana, Christopher M Reid, Alice J Owen, David P S Fong, Usha Gowda, Andre M N Renzaho

**Affiliations:** Centre for Cardiovascular Research and Education in Therapeutics, Department of Epidemiology and Preventive Medicine, Monash University, The Alfred Centre, Melbourne, Victoria Australia; Doutta Galla Community Health Service, Kensington, Victoria Australia; Global Health and Society Unit, Department of Epidemiology and Preventive Medicine, Monash University, Clayton, Australia; Centre for International Health, Department of Epidemiology and Preventive Medicine, Monash University, Burnet Institute, Melbourne, Victoria Australia; School of Social Sciences and Psychology, University of Western Sydney, Sydney, New South Wales Australia

**Keywords:** Vitamin D deficiency, Migrants, Cardiovascular diseases, Framingham 10 year risk score

## Abstract

**Background:**

Vitamin D deficiency is a global public health problem associated with increased risk of cardio-metabolic diseases and osteoarthritis. Migrants with dark skin settled in temperate climates are at greater risk of both vitamin D deficiency and cardiovascular diseases. This study aims to identify the risk of vitamin D deficiency and associations with cardiovascular disease in a migrant population in Australia.

**Methods:**

An audit was carried out at a Community Health Service in Kensington, Melbourne which, services a large migrant population. Data from the clinical records of all adults who visited the medical centre at least once during the period from 1^st^ January 2010 to 31^st^ December 2012 was extracted. The future (10 year) coronary heart disease risk was estimated using Framingham Risk Score.

**Results:**

The centre has given higher priority to vitamin D testing in migrants, those middle-aged, females and those with diabetes and osteoarthritis. Migrants from countries located in lower latitude regions (Latitude N23^0^ to S23^0^) were 1.48 (95% C.I. 1.32-1.65) times more likely to develop vitamin D deficiency post migration and 0.44 (95% C.I. 0.31-0.62) times less likely to have a >15% 10-year risk of coronary heart disease when compared to their Australian-born counterparts.

**Conclusions:**

Adherence to a high risk strategy for vitamin D testing was observed in the centre. Pre-migration latitude is an important factor for vitamin D deficiency (lower the latitude higher the risk) and in predicting future risk of cardiovascular disease in migrants. These findings suggest that a targeted approach for vitamin D testing, including zone of origin might better identify individuals at higher risk of both vitamin D deficiency and cardiovascular disease.

## Background

Vitamin D deficiency (VDD) has been classified as a pandemic affecting more than one billion people across all ages and ethnic groups worldwide [1]. An internationally standardised cut-off for deficiency is still under debate, but it is commonly defined as serum 25-hydroxyvitamin D (25(OH) D) levels less than 50 nmol/L, with levels of 50–75 nmol/L considered as borderline insufficiency [2, 3]. There are few rich sources of vitamin D in food and the main source for humans is the conversion of pro-vitamin D (7-dehydrocholesterol) to pre-vitamin D3 following skin exposure to ultraviolet B (UVB) radiation, which is subsequently converted to vitamin D3 via a heat dependant process [4]. Most people can meet their vitamin D needs through exposure to sunlight [4], however synthesis of vitamin D varies by colour of the skin (determined by the amount and type of melanin) in addition to the extent of exposure.

Despite the fact that Australia has a relatively sunny climate the prevalence of VDD (mean serum 25(OH) D < 50 nmol/L) has been estimated as 31% (females - 39% males- 22%) and at high as 50% during winter-spring [5]. Prevalence of vitamin D insufficiency (VDI) (mean serum 25(OH) D < 75 nmol/L) was reported to be 73%. Risk factors identified for VDD in Australia are older age, female gender, non-European origin, obesity, physical inactivity and higher level of education. In migrant populations risk factors included darker skin colour, Muslim religion, longer length of stay in Australia, full-covering clothing, decreased daylight exposure, testing vitamin D levels during winter or spring, living in an urban environment, coming from a socio-economically disadvantaged background and being an inpatient or institutionalised [5–7].

Migrants from low- and middle-income countries, particularly from South East Asia, the Middle East and Africa, constitute a significant segment of the Australian population. The latest Australian census showed that 27% of the resident Australian population was born overseas, an increase of 23.1% from 2001 [8]. Studies elucidating the prevalence of VDD among these populations reported values as high as 88% [9] and an understanding of the burden of disease associated with VDD in these at-risk sub-populations are lacking. Migration from low- and middle-income countries to industrialised countries has been found to be associated with increased risk of many chronic diseases [10]. While changes in lifestyles and acculturation are thought to be significant drivers of chronic disease risk, they do not solely explain the exponential risk of metabolic diseases in these sub-populations. It is possible that VDD plays a role in increasing risk of cardiovascular diseases (CVD).

Darker skin types of the some immigrant populations while offering protection against the intensity of sunlight in their countries of origin become a risk factor for VDD when relocating to countries further from the equator [11]. The origin of migrants could be classified into three zones according to ability, to synthesize adequate vitamin D as identified by Arabi et al. [12]. This has been developed using the country specific vitamin D levels.

- ● Zone 1 - sufficient Vitamin D production year-round
- ● (Latitude N23.5° to S23.5°)
- ● Zone 2 - insufficient Vitamin D production at least one month/year
- ● (Latitude N23.5° to N48° and > S23.5°)
- ● Zone 3 - insufficient Vitamin D production most of the months/year (Latitude > N48°)

VDD has been long been associated with bone diseases such as rickets, osteomalacia and osteoporosis, but recent studies also suggest a relationship between VDD and cardiovascular risk factors [13], including hypertension, diabetes, and metabolic syndromes [14]. What pre-migration factors influence vitamin D status and chronic diseases post migration is not clear, however studies suggest that those who have Vitamin D levels less than 37 nmol/L and hypertension have double the risk of developing myocardial infarction, heart failure or stroke compared to those with Vitamin D levels more than 37 nmol/L [15].

A number of algorithms for estimating cardiovascular risk, however the Framingham risk score (FRS) is a widely accepted method to estimate the risk of coronary heart disease (CHD) [16]. The method of calculation of FRS is given in detail elsewhere [17]. FRS has been used in Australia by the National Vascular Disease Prevention Alliance to assess the absolute CVD risk [18], and Zomer and colleagues validated FRS to Australian population by using a large countrywide sample [19].

With a large and ethnically diverse population living in Australia, information on CHD risk factors in migrant sub-groups are limited [20]. Therefore, we hypothesised that:

- ● Migrants will be more likely to be tested for vitamin D than Australian-born residents
- ● Migrants from sufficient vitamin D zones prior to migration will be at increased risk of VDD post-migration
- ● Migrants from sufficient vitamin D zones prior to migration will have reduced risk of CHD than Australian born residents
- ● VDD will be associated with a higher 10 year cardiovascular risk, and this association will vary by migration status

## Methods

### Setting and data collection

This cross sectional clinical audit was carried out in a Community Medical Centre in Kensington, Melbourne, Australia, which services a large migrant population and has a specific refugee health program. Moonee Valley and Melbourne City were the two main local government areas of this centre, and census data indicate that in those areas the proportion of residents born overseas is 28.8% and 49.4% respectively [21, 22]. Electronic medical records were accessed and the most recent laboratory test results for vitamin D levels, risk factors for CHD, and demographic data, including migrant status, county of origin were collected while maintaining patient anonymity. The blood pressure reading of the last visit to the clinic was used to identify the individuals as hypertensive (>140/90 mmHg).

### Inclusion and exclusion criteria

**Figure 1:**
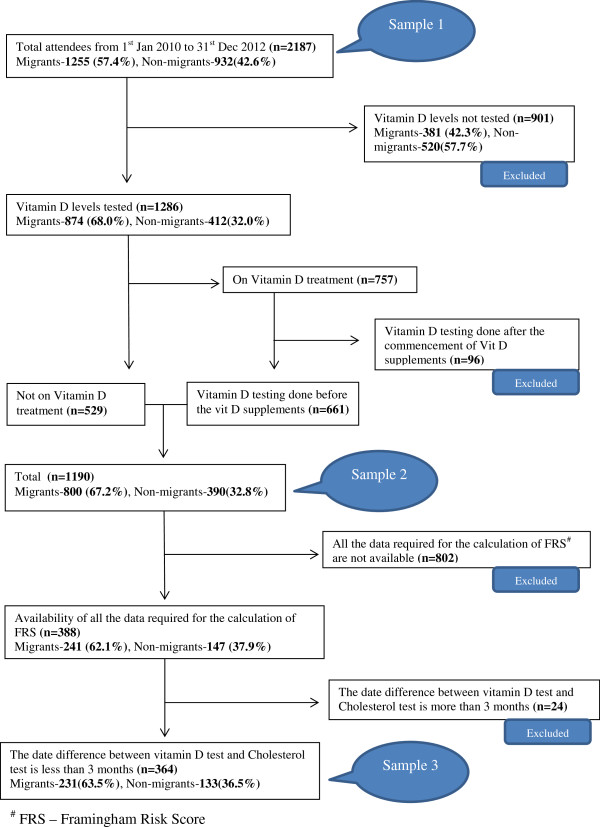
**Sampling method.**

Adults (19–99 years) (both migrants and non-migrants) who visited the medical centre at least once during the period from 1^st^ January 2010 to 31^st^ December 2012 (three years) were included in the study. The information in the last visit was used for those who visited more than once during the specified period. Data cleaning was undertaken and three population samples were drawn at different levels for different purposes. (Figure 1) Sample 1 was used to study the vitamin D testing pattern of the centre, while sample 2 was used to associate the country of origin latitude to the post migration VDD. The association between VDD and the FRS was assessed using the third sample. Steps were taken to eliminate the effect of supplementation on vitamin D test results in sample 2 and 3 (Figure 1).

### Data analysis

The correction method (“log (vit D) + [overall mean log (vit D) – mean (log vit D) sampled in same month])” described by Tomson et al. [23] was used to adjust for seasonal variation of the vitamin D levels in the cohort. Serum vitamin D (25(OH) D) level < 50 nmol/L was considered as deficient. Data was summarised using descriptive statistics. Log binomial regression method was undertaken to test the association between VDD, cardiovascular risk factors, cardiovascular risk score, and the zone of origin of the migrants. Data were analysed using IBM SPSS version 21. The study was approved by the Monash University Ethics Committee (CF12/3788 – 2012001835).

## Results

### Vitamin D testing pattern of the community medical centre (*sample 1*)

The total population (of whom 59% were migrants) was used to examine the vitamin D testing pattern of the centre. Data presented in Table 1 suggest that, after controlling for age and gender, the centre has given higher priority in testing vitamin D levels in migrants from zone 1 and 2, those who are middle-aged, females, and those with diabetes, or osteoarthritis.

**Table 1:** **Vitamin D testing pattern (assessed by the prevalence ratios) of the institution (n = 2187) during the specified period**

	Vitamin D tested 1287 (58.8%)	UPR ^#^(95% C.I.)	APR ^#^(95% C.I.)
**Migration** ^$^			
Non-migrant	412 (44.2)	*	*
Migrant-Zone 1	518 (74.3)	**1.68** (1.54-1.82)**	**1.65** **(1.51-1.79)**
Migrant-Zone 2	310 (66.0)	**1.51** **(1.37-1.65)**	**1.44** **(1.30-1.59)**
Migrant-Zone 3	46 (52.3)	1.13 (0.89-1.42)	1.12 (0.89-1.41)
**Age groups** ^$$^ **(Y)**			
<44	745 (54.6)	*	*
45-64	363 (64.6)	**1.18** **(1.09-1.28)**	**1.16** **(1.08-1.24)**
>65	178 (68.2)	**1.25** **(1.13-1.37)**	1.15 (0.99-1.26)
**Gender** ^$$$^			
Males	524 (54.4)	*	*
Females	762 (62.3)	**1.14** **(1.07-1.23)**	**1.07** **(1.02-1.14)**
**Employment** ^@^			
Employed	381 (54.8)	*	*
Unemployed	905 (60.7)	**1.11** **(1.02-1.20)**	0.99 (0.92-1.07)
**Smoker** ^@^			
No	289 (54.0)	*	
Yes	997 (60.4)	**1.12** **(1.02-1.22)**	0.99 (0.91-1.07)
**Drinker** ^@^			
No	480 (52.5)	*	
Yes	806 (63.4)	**1.21** **(1.12-1.30)**	1.02 (0.94-1.10)
**Diabetes** ^@^			
No	1183 (57.5)	*	*
Yes	103 (80.5)	**1.40** **(1.27-1.54)**	**1.30** **(1.16-1.45)**
**CHD** ^@^			
No	1240 (58.4)	*	*
Yes	46 (74.2)	**1.27** **(1.09-1.48)**	1.14 (0.98-1.34)
**Osteoarthritis** ^@^			
No	1197 (57.5)	*	*
Yes	89 (83.2)	**1.44** **(1.31-1.58)**	**1.29** **(1.17-1.43)**

^#^UPR – Unadjusted Prevalence Ratio, APR – Adjusted Prevalence Ratio.

*Reference category.

**Bold numbers – Statistically significant at 0.05 level.

Adjusted for - ^$^-Age and Gender, ^$$^-Latitude and Gender, ^$$$^-Latitude and Age, ^@^-Latitude, Age and Gender.

### Association between pre-migration latitude and post-migration VDD (*sample 2*)

Further analysis using a subset of the cohort (sample 2) was used to examine the associations between pre-migration latitude and VDD, eliminating those using vitamin D supplements. VDD in migrants from three identified pre-migration zones were compared to those born in Australia. As shown in Table 2, migrants from Zone 1 (sufficient Vitamin D production year round) were approximately 1.5 times, and those from Zone 2 (insufficient Vitamin D production at least one month per year) were almost 1.2 times, more likely to be VDD than Australian-born residents after adjusting for age and gender. This suggests that the lower latitude, the higher the prevalence of VDD in migrants relocating to Australia.

**Table 2:** **Vitamin D deficiency and country of birth of the migrants (n = 1190)**

Country of birth	VDD ^@^(%)	UPR ^**^(95% C.I.)	APR ^**^(95% C.I.)#
Born in Australia*	201 (51.5)	*	*
**Zone 1** (Latitude N23^0^ to S23^0^)	357 (75.2)	**1.48** (1.32-1.65)***	**1.46** **(1.31-1.63)**
**Zone 2** (Latitude N23^0^ to N48^0^ and > S23^0^)	171 (61.1)	**1.17** **(1.03-1.34)**	**1.14** **(1.00-1.30)**
**Zone 3** (Latitude > N48^0^)	21 (46.7)	0.92 (0.65-1.29)	0.91 (0.66-1.25)

^@^Vitamin D deficiency.

*Reference population.

**UPR – Unadjusted Prevalence Ratio, APR – Adjusted Prevalence Ratio.

***Bold numbers – Statistically significant at 0.05 level.

^#^Adjusted for Age and Gender.

### Association of zone of origin with cardiovascular risk

Migrants from Zone 1 showed lower smoking rates and higher cholesterol/HDL ratios, but were 0.44 likely to have a >15% risk of developing future (10 year) CHD events when compared to their Australian-born counterparts (Table 3).

**Table 3:** **Zone of origin and its association with post migration CHD** ^**$**^ **risk (n = 364)**

Reference group		Migrants (n = 231, 63.5%)			
Those who born in Australia (n = 133, 36.5%)		Zone 1 (n = 119)		Zone 2 + 3 (n = 112)	
n (%)		(Latitude N23 ^0^to S30 ^0^)		(Latitude > N23 ^0^and > S30 ^0^)	
		n (%)	APR ^@^(95% C.I.)	n (%)	APR (95% C.I.)
**Hypertension** ^*^ **(>140/** **90 mmHg)**	22(16.5)	16 (13.4)	0.86 (0.44-1.66)	26 (23.2)	1.50 (0.89-2.52)
**Current smoking** ^**^	66(49.6)	15(12.6)	**0.25** (0.14-0.44)***	29(25.9)	**0.59** **(0.41-0.87)**
**Diabetes** ^**^	22 (16.5)	23 (19.3)	1.36 (0.79-2.37)	24 (21.4)	0.98 (0.56-1.73)
**Cholesterol** ^**^ **(mmol/L)**					
% High cholesterol level (>5.5)	51 (38.3)	44 (37.0)	0.91 (0.66-1.26)	37 (33.0)	0.81 (0.56-1.17)
% of HDL (<1.0- men, <1.3-women)	62 (46.6)	58 (48.7)	1.22 (0.94-1.59)	47 (42.0)	1.09 (0.83-1.43)
Cholesterol/HDL ratio >4.5	34(25.6)	43(36.1)	**1.64** **(1.12-2.40)**	32 (28.6)	1.43 (0.95-2.14)
**Coronary heart disease** ^**^	8(6.0)	2 (1.7)	0.57 (0.12-2.65)	10 (8.9)	1.20 (0.43-3.24)
**FRS** ^#^ >**15**%	74 (55.6)	29 (24.4)	**0.44** **(0.31-0.62)**	59 (52.7)	0.95 (0.75-1.19)

$*CHD* – Coronary heart disease.

^@^ *APR* – Adjusted Prevalence Ratio.

*Adjusted for Age, Gender, Smoking, Diabetes, Cholesterol/HDL ratio, Vitamin D (serum 25(OH) D).

**Adjusted for Age and Gender.

***Bold numbers – Statistically significant at 0.05 level.

^#^Framingham Risk Score - Adjusted for Vitamin D (serum 25 (OH) D) only as this is a composite index of Age, Gender, Smoking, Diabetes, Cholesterol/HDL ratio, Hypertension. The cut off level was arbitrarily set at 15 (mean – 16.3 and median – 12.7).

Pre-migration zone 3 (n = 12) was amalgamated to zone 2 due to its small numbers.

### Association between VDD and the risk of CHD by migration status

In those for whom 10-year Framingham risk score could be calculated, there was no association between VDD and cardiovascular risk in migrants or non-migrants (Table 4).

**Table 4:** **VDD** ^**@**^ **as a risk factor for higher 10 year CHD** ^**^ **risk score (n = 364)**

			FRS ^$^>15 (%)	UPR ^#^(95% C.I.)	P value
**Migrants**					
Zone 1 (n = 119)	VDD	No	7 (26.9)	1.00*	
		Yes	22 (23.7)	0.92 (0.44- 1.92)	0.83
Zone 2 (n = 112)	VDD	No	11 (40.7)	1.00*	
		Yes	48 (56.5)	1.32 (0.81- 2.14)	0.26
**Non-** **migrants** (n = 133)					
	VDD	No	25 (59.5)	1.00*	
		Yes	49 (53.8)	0.91 (0.66-1.24)	0.53

**Coronary heart disease.

*Reference level.

^$^Framingham Risk Score - No adjustments were made as FRS is a composite index and the cut off level was arbitrarily set as 15 (mean – 16.3 and median – 12.7).

^#^UPR – Unadjusted Prevalence Ratio.

^@^VDD – Vitamin D Deficiency.

## Discussion

This study examined vitamin D testing patterns among a multicultural sample of Australian adults in north-east Melbourne, Australia and examined whether pre-migration latitude was a risk factor for vitamin D deficiency and cardiovascular risk post-migration. Migrants, especially those from Zone 1 and 2, were more likely to be tested for vitamin D than their Australian-born counterparts. However given the criteria set for data extraction by the audit it cannot be determined to what extent the decision to test for vitamin D testing is informed by clinical decisions. It is possible that the preferential vitamin D testing among migrants is simply driven by practitioners’ perceived risk of VDD among migrant populations. Current evidence suggests that the cost of vitamin D testing in Australia has increased from $1.02 million in 2000 to $95.6 million in 2010, representing a 94-fold increase over a 10-year period [24]. Current Australian recommendations [25] are to test those at increased risk of VDD, such as elderly and those with darker skin. Formal guidelines that govern the timing of testing (diagnosis) and frequency (monitoring) of vitamin D testing and VDD treatment are urgently needed [26].

In the present study cohort vitamin D testing was also more frequently undertaken in middle-aged people, females and those with chronic disease indicative of a testing pattern adhering to the high risk approach [27]. This finding is supported by previous analysis of data from the national health care provider, Medicare Australia, showing the majority of the vitamin D test had been performed in middle aged adults (30–64 years) and in females [28].

The findings of this study suggest that those migrating from a zone where sunlight is sufficient to achieve year-round vitamin D production (closer to the equator) are at increased risk of VDD post-migration. Migrants from Zone 1 generally have a darker skin which acts as a barrier for the skin penetration of the UVB rays and may reduce the production of Vitamin D [29]. Our findings are consistent with the literature showing the higher risk of VDD in refugee population who migrated to Australia from countries closer to the equator [30]. For example, studies done among immigrants from Africa to Australia reported a prevalence of VDD as 53% (men 20%, women 74%) [31] compared to the general population, which was 31% (men 22%, women 39%) [5].

The present study also found that those migrating from areas with sufficient vitamin D all year to a southern Australian environment had a lower CHD risk than those who born in Australia. A study looking at ischemic heart disease, place of birth and, language, utilizing the Australian national hospital morbidity database, found that migrants were at higher risk for cardiovascular diseases [32]. However, the Australian Institute of Health and Welfare has consistently reported that those who born overseas experienced a lower CHD specific mortality rate compared to those who born in Australia [33–35]. Possible reasons for this could be the mandatory health checks imposed pre-migration by the authorities and/or the genetic factors. Although Australian immigration, health checks do not specifically test for CHD [36], it does include a general health screening. As a result of this healthier individual get the opportunity to settle, and this effect has been termed the “healthy migrant effect” [35]. However if post-migration, this group are at increased risk of VDD and its long-term sequel, further research is needed to discern what are the key factors driving this increased risk of VDD, which may include cultural, socioeconomic and genetic factors.

The prevalence of CHD in Australia has been reported to be 3.0% (males 4.4%, females 2.3%) [37] and the mean 10-year Framingham cardiovascular risk to be 6.3% [38]. Observational studies have suggested an association exists between vitamin D status and cardiovascular disease outcomes [39]. A meta-analysis in 2012 found a linear inverse association between vitamin D (ranging from 20–60 nmol/L) and cardiovascular disease. They reported a small but significant increase in risk of 1.03 (95% CI 1.00, 1.06) with vitamin D deficiency [13]. While studies suggest an inverse association between vitamin D and vascular and non-vascular mortality, the causality is still unclear [23]. The present study found no association between VDD and cardiovascular risk as estimated by the Framingham risk score (which is derived from a set of long established risk factors; age, sex, plasma cholesterol, blood pressure, diabetes and smoking status). Weak associations between vitamin D and both plasma cholesterol and blood pressure have been suggested by some [40, 41], but certainly not all [42, 43], studies. Whether the influence of VDD on cardiovascular disease risk might be driven via those traditional risk factors or other factors remains unclear.

### Strength and limitations

The retrospective data collection method used in the present study was preferred over the prospective data collection to avoid biasing vitamin D testing pattern of the centre. The cohort size was substantially reduced for examination of cardiovascular risk due to lack of availability of risk factor data for some patients. Cholesterol testing within 3 months of vitamin D testing date was used for analyses. As subcutaneous vitamin D synthesis depends on the solar ultraviolet B radiation, there were, as expected, marked seasonal differences [44]. As this study used vitamin D results collected throughout the year, a seasonality adjustment was undertaken. While body mass index (BMI) is an important determinant for both vitamin D status [45] and CHD [46] it was not available for a sufficient proportion of the cohort to be used in the analyses undertaken in this study.

Post-migration duration in Australia is an important variable which was not available for this cohort. According to Young [47], the “healthy migrant effect” diminishes incrementally with the number of years stay post-migration. The absence of this data is a limitation in the current analysis. In addition, migrants were categorised in pre-migration zones by country of origin. As some larger countries may belong to more than one zone, this could lead to a misclassification. Other limitations include lack of information available for use of full-cover clothing, and that those attending medical centres may be different from the general population. Details related to the laboratory methodology for vitamin D testing was also unavailable to the audit and is acknowledged as a limitation. The lack of association between VDD and Framingham risk seen in the present study should be interpreted with caution, given the limited sample size. However the study represents a contemporary picture of vitamin D testing patterns and cardiovascular risk in a public health clinic servicing a large migrant population.

## Conclusions

The community based medical centre demonstrated adherence to a high risk strategy for vitamin D testing, with higher priority given to migrants, middle aged adults and females. An increasing risk of VDD in those who migrated from lower latitudes (closer to the equator) was observed. However migrants had lower CHD risk compared to those who born in Australia. This audit suggests that pre-migration zone may be an important factor relating to the future risk of CHD (the lower the latitude of origin the lower the future CHD risk), and may be due to a low smoking rate seen among migrants. Zone of origin should be considered in the development of vitamin D testing guidelines. We found no evidence of an association between VDD and CHD risk in this cohort, but given the multifactorial risk and long latency of cardiovascular disease, longitudinal studies may be required to examine this further.

### Recommendations and policy implications

The current study has identified the high prevalence of vitamin D deficiency in migrant populations classified according to their pre-migration zones, and its association with some of the cardiovascular risk factors. In light of the significant burden of vitamin D deficiency, sun exposure, vitamin D testing and supplementation guidelines should consider disadvantaged groups including migrants and their zone of origin.
